# Helping People Through Space and Time: Assistance as a Perspective on Human-Robot Interaction

**DOI:** 10.3389/frobt.2021.720319

**Published:** 2022-01-27

**Authors:** Benjamin A. Newman, Reuben M. Aronson, Kris Kitani, Henny Admoni

**Affiliations:** Robotics Institute, Carnegie Mellon University, Pittsburgh, PA, United States

**Keywords:** human robot interaction, assistive robotics, socially assistive robotics, physically assistive robotics, collaborative robotics, rehabilitative robotics

## Abstract

As assistive robotics has expanded to many task domains, comparing assistive strategies among the varieties of research becomes increasingly difficult. To begin to unify the disparate domains into a more general theory of assistance, we present a definition of assistance, a survey of existing work, and three key design axes that occur in many domains and benefit from the examination of assistance as a whole. We first define an assistance perspective that focuses on understanding a robot that is in control of its actions but subordinate to a user’s goals. Next, we use this perspective to explore design axes that arise from the problem of assistance more generally and explore how these axes have comparable trade-offs across many domains. We investigate how the assistive robot handles other people in the interaction, how the robot design can operate in a variety of action spaces to enact similar goals, and how assistive robots can vary the timing of their actions relative to the user’s behavior. While these axes are by no means comprehensive, we propose them as useful tools for unifying assistance research across domains and as examples of how taking a broader perspective on assistance enables more cross-domain theorizing about assistance.

## 1 Introduction

Smart wheelchairs navigating easily through crowded rooms, coaching robots guiding older adults through stroke rehabilitation exercises, robotic arms aiding motor-impaired individuals to eat a meal at a restaurant: these are all examples of research in areas as disparate as intelligent motion planning, rehabilitative medicine, and robotic manipulation that have been independently identified as being able to contribute to the development of robots that can do helpful things for people. This research has been fruitful, but has remained siloed as researchers from these various fields focus on the specific assistive tasks relevant to their own disciplines.

A lack of common structure in the field of assistive robotics makes it difficult for researchers to incorporate findings from other domains into their own work. For example, how does the relationship between a grocery stocking robot and the surrounding customers relate to the relationship between an airport guide robot and the surrounding crowd? Does a robot designed to autonomously declutter a room convey a similar sense of agency as a virtual robot suggesting an optimal ordering in which you should clean your room? Answers to these and similar questions would form a basis that would provide clarity for research in assistive robotics, but are currently difficult to determine due to the disparate nature of assistive robotics.

In this work, we identify a subset of common challenges and develop themes that begin a conversation about how assistance abstracted from specific problem domains and can be used to answer questions about assistance generally, thereby benefiting the entire field of assistive robotics. This would enable researchers to explore the underlying principles of assistive robotics and communicate them across domains. To start, we suggest that assistance is not a characteristic of a robotic system as it has been historically treated. Instead, assistance is a task-independent perspective on human robot interaction. Treating assistance as a task-independent perspective on HRI, we can group existing assistive research by its effect on three key axes: people (e.g., who is involved in the system and the roles they play), space (e.g., how the robot’s action affects the task), and time (e.g., when the robot performs its actions during the task).

This perspective considers an assistive system as an interaction in which a user and a robot forge a complex, asymmetric relationship guided by the user’s goals. This perspective is somewhat different from general HRI because the user is responsible for determining the interaction’s end goal while the robot acts in service of this goal. Similar to other collaborative settings, the human-robot pair is then tasked with performing subsequent actions to achieve the human’s goal, but unlike some collaborations, maintaining human autonomy is paramount. In this relationship, the robot has more agency and independence of action choice than a simple tool (i.e., the robot’s choice of action is not determined solely by the user), but it must defer to the user’s goal and independent actions.

We introduce three design dimensions with which roboticists can begin to reason about the assistive interactions of robots and humans. First, we discuss how the assistive robot’s role can be described with respect to the relationship it has with its user, for example, how it weighs priorities when there are multiple potential people it could assist. Second, we propose that an assistive robot’s role can be described in terms of how it operates in the execution space, that is, the space in which the robot has its primary effect. Finally, we propose that the same robot’s actions can be described in terms of the temporal space, that is, the duration and sequence of the actions. We support these dimensions by reviewing and grouping over 200 recent assistive robotics research papers.

By using assistance as a lens through which to analyze patterns that arise in assistive robotics, we hope to help designers of assistive robots more easily explore the design space and identify similar examples of past solutions, even across application domains. Additionally, we hope this work will motivate researchers to continue to refine this notion of assistance and its effects on human-robot interaction paradigms.

## 2 The Assistance Perspective

In the field of robotics, defining assistance can be tricky. In a broad sense, every robot is built to assist some person. Therefore, we do not attempt to separate assistive systems from non-assistive systems. Instead, we propose assistance as a particular perspective through which many robotic systems can be viewed. This perspective considers robotic agents that are autonomous in action but subordinate in goal to a human partner. Almost any robot system can, in theory, be viewed as assistive to someone, so we do not limit this scope. Rather, we explore what this analytic framework provides. This perspective clarifies particular design tradeoffs and trends general to assistive systems whatever their task domain. In this work, we describe several key design axes that arise when considering a robotic system as assistive and discuss implications these axes have on the interaction.

Before discussing these key design axes, we first formalize what we mean by a human-robot interaction, then provide a more detailed description of what it means to view assistance as a perspective. Next, we give a brief synopsis of previous attempts to characterize assistance and assistive robotics, and finally we give an overview of the remainder of this paper.

### 2.1 General Human-Robot Interaction

Before discussing assistance, we first sketch a general framework for human-robot interaction, which we draw broadly from multi-agent systems research. Formalizations of this problem can be found in previous literature (Jarrassé et al., 2012); here we only establish enough language to discuss assistance rather than requiring assistive systems to use this exact model.

First, we define a user *u* ∈ *U* as any person involved closely in the interaction. Typically, the user is in close physical proximity to the robot and provides explicit or implicit control signals to the robot. For example, a person teleoperating a robotic arm, getting directions from a social robot, or building a table with a robot helper, would be considered a user.

Next, the system has at least one robot *r* ∈ *R*. Canonically, a robot is defined as an embodied system that can sense its environment, plan in response to those sensory inputs, and act on its environment. An assistive robot may have a wide array of sensory, planning, and acting capabilities in order to be successful in its task. Some of these capabilities will be critical for the robot’s functioning (e.g., LIDAR to avoid hitting obstacles), while others will be critical for providing assistance to the user (e.g., a body pose recognition algorithm to identify the user’s location and gestures).

Finally these agents exist in a shared environment, each with its own internal state. These are described in totality by the mutual state *s*_*m*_ = (*s*_*r*_, *s*_*u*_, *s*_*e*_) that defines the individual states of the robot, user, and environment. The robot and user both have goals *g*_*r*_, *g*_*u*_ ∈ *G* and can take actions *a*_*r*_ ∈ *A*_*r*_ and *a*_*u*_ ∈ *A*_*u*_ that affect their mutual state. By acting to update their mutual state, each agent has the potential to affect the other agent’s behavior resulting in an interaction between the two agents. Depending on the exact scenario, a task will be considered complete when one or more agents has achieved their goal.

### 2.2 Assistance as a Perspective on Human-Robot Interaction

Using this formulation, we can more carefully define assistance. Assistive systems interpret the robot as autonomous in its actions but subordinate in its goal. By giving the user the sole responsibility for setting both agents’ goals, the two agents now attempt to satisfy some shared goal *g* by reaching a mutual state where *g* is true: $s_{m}^{g}$ . This framing distinguishes assistive robotics from both traditional assistive technologies like a white cane, which has no control over its actions or goals, and traditional robotics, which develops systems with full control over their actions and goals. This framing gives rise to three key design axes: how assistive robots affect *people* through *space* and *time*. The discussion of these implications is the subject of the rest of this paper.

In HRI, as in assistive robotics, there is no requirement for there to be a single user. In fact, many assistive robotics scenarios involve more than one user. This becomes challenging, as it is the responsibility of one of these users to set the goal for the robot, but selecting which user has this responsibility may change the type of assistance the robot is able to provide. This is especially true when one user’s goals may conflict with another user’s goals. This highlights the importance of determining the roles of people when considering assistive robotics problems (Section 4).

Furthermore, since the user and robot are working to accomplish the same goal, the robot has freedom over its action space. As a baseline, the robot can assume the user would perform the task independently, without its aid. The robot can then choose its action space to align with how it can most beneficially assist the user over this baseline scenario. In addition to the standard strategy of directly manipulating the environment, the robot can assist by altering the user’s state space, encouraging the user to make more effective task progress. For example, a head-mounted augmented reality device displaying the optimal path for cleaning a room can assist the user without needing to physically interact with objects. Assistive scenarios allow more choice over the robot’s action space than would a general robot (Section 5).

Finally, in order to advance to the mutual goal state and complete the task, the user and robot each complete a sequence of actions ( $a_{u}^{1},\ldots ,a_{u}^{t}$ , $a_{r}^{1},\ldots ,a_{r}^{t}$ , respectively) that transition the system to the desired goal state $\left(s_{m}=s_{m}^{g}\right)$ . Given that these actions occur in the mutual state, it is important that the user and the robot time their actions appropriately, so that they do not attempt to solve the same part of the task simultaneously, or worse, provide conflicting actions that result in undoing each other’s work. How to time actions is crucial to studying assistive robotics (Section 6).

Each of these axes presents researchers with decisions that result in critical trade-offs when designing an assistive robot. Throughout the remainder of this work, we will describe how assistive robots from different application domains fall along these axes.

By taking assistance as a perspective, it is our goal to provide an abstraction that allows for comparing systems from different domains to discover universal challenges that arise from robot assistance. We do not suggest that these axes describe a full assistive system or are a complete set of critical design axes. Rather, viewing assistance along these particular axes of people, space, and time enables some cross-domain comparisons and insights on its own, and it also demonstrates how assistance overall can benefit from a general examination.

### 2.3 Prior Categorizations of Assistive Robotics

By grouping assistive robots along the aforementioned design axes, we view assistance as an abstract concept that illuminates parallel research problems across different application domains. We build on previous literature which categorizes assistive robotics within particular application domains, for example socially assistive robots (Fong et al., 2003; Matarić and Scassellati, 2016), joint action (Iqbal and Riek, 2019) and physically assistive robots (Brose et al., 2010).

Some work does try to describe assistance as a whole. Jarrassé et al. (2012) categorizes joint action between dyads by positing a cost function for each agent defined on each agent’s task error and required energy. Among categories in which both agents are working together towards the same goal, the paper specifies collaboration between two equal peers, assistance when one agent is subordinate to another, and education in which the educator assists the partner but moderates its own effort to encourage increasing effort from its partner. We take this core idea of assistance as subordination and build on it in our definition of the assistance perspective.

Most similar to the current work, perhaps, is the accounting given in Wandke (2005). This overview of assistance in human-computer interaction notes that defining assistance as any system that provides some benefit to the user would include nearly all technical artifacts. Therefore, the paper restricts its attention to systems that bridge the gap between a user and the technical capabilities of the system due to the user’s unfamiliarity with the system or excessive burden of use. In contrast to this approach, our work presents assistance as a perspective rather than a definition; it could in principle be applied to any technical artifact but may only be useful for some. Additionally, this definition of assistance focuses on how assistive systems correct a deficiency in a user’s understanding of the system or capability to use it. In contrast, our definition of assistance as a perspective admits beneficial actions from the robot of all sorts, not just those repairing the user’s ability to use a system.

### 2.4 Overview of This Paper

By defining assistance as a perspective, we provide language to discuss ideas about assistance from different domains. This will allow researchers from various areas of assistive robotics to come together to illuminate and discuss common research challenges. Additionally, researchers can make design decisions about how the assistive robot affects people in space and time by using this framework to consider similar approaches to problems from disparate task domains. In the remainder of this paper, we discuss these design axes and explore their implications through a review of existing assistive robotics literature. Section 3 describes our method for collecting these papers Section 4 describes the people design axis, Section 5 describes the space design axis, and Section 6 describes the time design axis. These axes are summarized in Table 1. We then conclude the paper with a discussion over the implications of this work.

**TABLE 1:** Assistive robots can be explored along three key axes: how the assistive system thinks about additional people, what part of the mutual state aligns with its action space, and at what time it executes its actions during a task.

Key axis	Description
**People** (Section 4)	How the robot considers additional people outside the baseline dyad.
Targets of assistance	Additional people whose goals are of comparable importance to the user.
Interactants	Additional people whose goals are not privileged and use general human-robot interaction approaches.
**Space** (Section 5)	The portion of the mutual state the robot’s actions affect.
Environment	The robot affects the environment directly by, e.g., manipulating task objects.
Human body	The robot affects the user’s body by physically moving some portion of their body.
Human brain	The robot affects the user’s mental state by providing information about the task or reducing the cognitive burden.
**Time** (Section 6)	The relative timing between a robot’s actions and the user’s explicit commands during the task.
Proactive	The robot acts before an explicit command.
Reactive	The robot acts in response to an explicit command.
Simultaneous	The robot acts simultaneously with user action.

## 3 Methods

To develop this taxonomy, we conducted a literature review of recent papers on assistive robotics.

### 3.1 Initial Search

First, we hand-selected 74 papers from the last 5 years of the annual Human Robot Interaction conference (HRI 2016–2020). From these papers we generated an initial set of search terms by aggregating titles, abstracts, and author generated keywords using the R (R Core Team, 2017) package litsearchr (Grames et al., 2019). Using these aggregated keywords, we formed an initial search query.

### 3.2 Refined Search

We ran the initial search query on the Web of Science. This search yielded approximately 1,500 papers. We repeated the keyword aggregation on this set of keywords, and then hand-selected new keywords from among them based on their prevalence and relevance to assistive robotics. We repeated the Web of Science query with this refined set of keywords, which yielded, again, approximately 1,500 papers. The refined search was run on 29th January 2021. We included a paper based on whether the following statement evaluated true based on a search of the entire text of the paper.

((assist^∗^ NEAR ^∗^robot^∗^)

OR (collab^∗^ NEAR ^∗^robot^∗^))

AND (^∗^human^∗^ OR ^∗^people^∗^ OR ^∗^person^∗^ OR ^∗^subject^∗^ OR ^∗^user^∗^ OR “elderly people” OR “older adults” OR “natural human” OR “stroke patients” OR “healthy subjects”)

AND (“human-robot interaction” OR “human-robot collaboration” OR “robot interaction” OR “robot collaboration” OR collaboration OR hri OR “human robot collaboration” OR “physical human-robot interaction” OR “human robot interaction” OR “machine interaction” OR “human-machine interaction” OR “human interaction”)

AND (“collaborat^∗^ task^∗^” OR “assembly task^∗^” OR “social interaction^∗^” OR “assembly process^∗^” OR “shared workspace^∗^” OR “manipulation task^∗^” OR “human safety” OR “daily living” OR “service ^∗^robot^∗^” OR “production system^∗^” OR “safety standard^∗^” OR “mobile robot^∗^” OR “assisted therap^∗^” OR “collision avoidance” OR “object manipulation” OR “collaborative assembly” OR “socially assistive” OR “assistive *robot^∗^” OR “social ^∗^robot^∗^” OR “teleoperat^∗^”))

### 3.3 Paper Selection

Starting from the refined Web of Science results, we filtered out all papers from venues with fewer than two related documents and papers that were older than 5 years, with a small exception. In an attempt to keep papers with significant contributions to the field, papers older than 5 years were kept if they had more than 10 citations. This process left approximately 465 papers. Each paper in this set was then manually checked for relevance by reading the title and abstract. To be included, we required the paper to include both 1) an assistive interaction with the user and 2) a system capable of taking actions. This step mainly removed papers focused on robotic system development or perception improvements rather than assistance itself. This yielded 313 papers, each of which was again reviewed against the aforementioned exclusion criteria. The entire search process yielded over 200 papers that we classified into our taxonomy.

## 4 People

**FIGURE 1:**
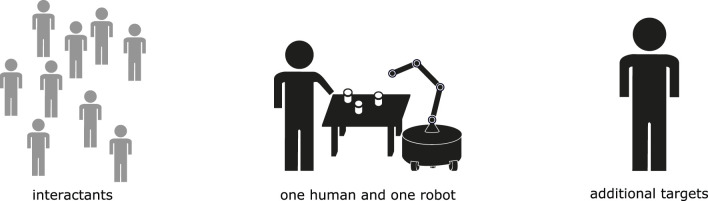
An assistive system can treat people beyond a single user as additional targets of assistance or as interactants, and either choice introduces particular complications into the assistive dynamic.

In Section 2, we described assistance with single users. This description works well for situations that have only one user, which is common in laboratory settings. In realistic settings, however, a robot will typically encounter more than one person in the course of completing their task. These other people can act in a variety of different roles within the interaction. In this section, we explore themes in how assistive interactions incorporate more people into the general human-robot dyad (Figure 1).

### 4.1 Terminology

The simplest approach a system can take towards other people is simply to ignore them completely. While this case tends not to be analyzed explicitly, it is implicit in many systems. This strategy can be appropriate, especially during situations in which encountering additional people is rare. When working with other people, though, the robot could implicitly account for additional people by relying on its primary user to provide controls that appropriately consider other users. Finally, a robot might intentionally downplay its relationship to additional people when accounting for them would conflict with its primary user’s goals, such as an emergency response robot that ignores standard social navigation behaviors to reach its patient as fast as possible.

When the system does choose to reason about other people, its treatment of them can be determined by dividing them into two different roles: the target of assistance, whose goals are of equivalent importance as other targets; and interactants, who require the attention owed to any other person as explored throughout human-robot interaction research but don’t have their goals privileged by the robot.

A target of assistance derives directly from the definition of assistance: an assistive scenario must support the goals of at least one person. Consider a scenario in which a person who has a spinal cord injury uses a robotic arm to aid them in eating a meal with friends at a restaurant. In this scenario, the arm’s user sets the goal for the robot: to bring food from their plate to their mouth so they can consume it.

The second role a person can play in an interaction is that of interactant. An interactant is any other person involved in the scenario who is not a target. Continuing the previous example, the people who are out to dinner with their robot-operating friend are interactants. They have no direct bearing on the robot’s goal, but they are potentially affected by the robot’s actions and may require some design effort for the system. For example, the robot may have to avoid collisions with them during its operation. While the robot’s relationship to interactants is not assistive, the presence of a specific target of assistance can affect how the robot interacts with others.

When considering assistive systems that involve more than a single target, the system must determine in which of these roles to consider the additional people. These two roles are not mutually exclusive; there can be more than one of each in a given scenario. Additionally, both targets of assistance and interactants can give explicit control input to the robot. Designating people as additional targets or as interactants brings about different challenges for the assistive system.

### 4.2 Additional Targets of Assistance

One challenge arising from a single robot having multiple targets of assistance is that the goals issued by these targets can conflict with one another. In the eating scenario, the robot might instead be assisting everyone present, perhaps by both feeding its user and serving food to other people at the table. Here, the robot is presented with a conflict: how should it choose to prioritize the goals given by its targets and reconcile differences between them?

This can be especially challenging in contexts such as education. An educational robot might consider the teacher as its target and work to enrich a student according to a mandated curriculum. It can also consider the student as its target and try to engage the student with concepts that are interesting to them regardless of the curriculum. Much research in this area aims to make the content proposed by the teacher more enjoyable by developing robotic behaviors that are meant to keep the student engaged. Leite et al. (2015) designed a robot puppet show to engage young learners in an educational story, Martelaro et al. (2016) designed a robot that encourages students to develop trust and companionship with their tutor, and Christodoulou et al. (2020) designed a robot to give nonverbal feedback to students in response to quiz answers to keep them engaged with the testing material. In contrast, Davison et al. (2020) took a different approach and developed the KASPAR robot to look like another student and deployed it in unsupervised interactions that were totally motivated by the student. In this way, they allowed the student to approach the learning material voluntarily, giving the student more agency to learn what they desired and at their own pace.

This dilemma can again be seen in therapeutic contexts, where a robot must reconcile the goals of the doctor and the patient. Robots can increase a patient’s motivation to do mundane, repetitive or uncomfortable exercises through the use of a robot that does the exercise alongside the patient (Tapus et al., 2007; Schneider and Kummert, 2016). Alternatively, a robot could be used to give the patient more agency and independence over their own treatment by helping someone independently practice meditation (Alimardani et al., 2020), do independent cognitive behavioral therapy (Dino et al., 2019), or home therapy for autism (Shayan et al., 2016).

A full analysis of these interactions treats both the teacher and the student, or both the therapist and the patient, as targets of assistance with goals that often align but are not identical. This alignment mismatch can often lead to ethical challenges, which are even more fraught when the capabilities, agency, and relative power of the possible targets vary. While there is no general technical solution, this language encourages designers to explicitly enumerate the multiple targets of the assistance and to reason directly about conflicts in their goals.

### 4.3 Additional Interactants

On the other end of the spectrum are robots that treat additional people in the system as interactants. Robots designed with this relationship in mind prioritize the goals of its target of assistance. In our assisted eating scenario, the robot may need to follow basic social norms around the other diners by avoiding collisions with them, but it does not privilege their goals.

This relationship is typically used in scenarios where some figure of authority (e.g., a teacher or a therapist) needs to relieve themselves of some amount of work. For example, a teacher could employ a robot to teach half of their class in order to reduce the student-to-teacher ratio for a particular lesson (Rosenberg-Kima et al., 2019), or even have the robot teach the class alone if they need to finish other work (Polishuk and Verner, 2018). In this way, the teacher is the target of assistance, while the students are treated only as interactants. The robot should be able to teach competently enough to achieve the teacher’s goals, but the students’ preferences about using the robot are not of direct concern.

Similarly in emotional or physical therapy a robot can be employed to lead group sessions in lieu of a doctor, who may have more classes than they can handle (Fan et al., 2016; Ivanova et al., 2017). Alternatively, the robot may be better at collecting certain information than the user. For example a patient who has suffered a stroke may be unable to emit certain social signals expected during social interaction. This could negatively affect a doctor’s opinion of this patient, a problem that could be circumvented by having a robot collect this information (Briggs et al., 2015; Varrasi et al., 2019). The patient here, however, is not asked whether they may prefer the social interaction regardless of the implicit bias the doctor may possess.

These systems don’t generally follow an assistance dynamic with interactants, rather, general human-robot interaction research applies. However, the fact that the system has a target, even if the target is not present, can change the robot’s behavior: a robot acting as a proxy for a specific teacher may have different behavior than one employed as a general-purpose robot, which might have bearing on how the general human-robot interaction problem is resolved.

### 4.4 Combinations of Roles

If an assistive robot has multiple additional people present in the interaction, it can choose to consider some of them as targets and others as interactants. In this relationship, our assisted eating robot might treat both the user and the companion seated next to them as targets of assistance, while those eating companions seated further away from the user are treated as interactants. In this way the robot can carefully maintain the goals of multiple people in proximity to the robot. This framework can allow for more complex robot behavior near to the user without the additional complication of handling everyone else at the table.

Another example would be a robot that participates in a collaborative scenario with multiple human actors, some of whom serve as both targets of assistance and interactants, while others are only interactants. For example, consider a local repair-person who needs help from a remote repair person. To give instructions, the remote repair person can use a robot to highlight the parts of the environment they are discussing (Machino et al., 2006). In this way, both actors are interactants in the scenario, but only the local repair person is the target of assistance.

### 4.5 Implications

These various relationships clarify the design choices involved in developing an assistive system. A particular task, such as assistive eating, does not require a particular relationship between the robot and the people it encounters. Rather, how a robot relates to these people is a design decision that will have implications as to how the task is completed.

The choice of roles affects how assistive systems with multiple people are evaluated. When treating the user and their eating companions all as targets of assistance, the robot would need to verify that it is helping them all in achieving their independent goals. This type of evaluation may be difficult to actually measure and nearly impossible to succeed on, as the companions have conflicting interests from the user. Identifying what type of relationship the robot should have with its users can help researchers disambiguate otherwise similar systems to determine which evaluations are important.

The choice of which roles to use may also have implications on how much autonomy to imbue in the robot. A robot that balances the goals of many people may require complex sensing, modeling, and planning to carefully moderate between them. A simpler robot might delegate this goal moderation problem to its user and treat additional people as interactants or ignore them entirely. This system gives the target more control over the goals, but requires additional input from the user. If the robot maintains full autonomy in this scenario, but it does not plan for other people’s goals, it may in fact endanger them by running into them where another system would have chosen to avoid them. These ideas show how the choice of relationship between the robot and the people it encounters throughout a task can impact the design of the final system.

## 5 Space

Assistive robotic systems can perform similar tasks by acting in different action spaces. We show in Section 2 how to represent the mutual state during the interaction as the state of the user *s*_*u*_, the state of the robot *s*_*r*_, and the state of the environment *s*_*e*_. In general, a user employing an assistive robots is aiming to make some alteration to *s*_*e*_. Since the robot is tasked with aiding the user and not directly accomplishing this state alteration, the robot can assist the user by making a change to any part of the mutual state that makes it easier for the user to accomplish their goal. In this maner, a robot can provide many different types of assistance when helping to complete the same overall task.

Consider an assistive eating robot. The robot and its user sit at a table across from one another, with a plate of food between them. The user’s goal is to eat the food. The robot can provide assistance by performing a variety of different actions: it can act on the user’s mental state by projecting a light onto a morsel of food that would be easy to grab next, it can change the physical state of the user by guiding their hand into an appropriate position, or it can change the environment by picking up the morsel and feeding it to the user. All of these action spaces apply to the same task and the same goal; what differs is in what way the user would most benefit from assistance.

**FIGURE 2:**
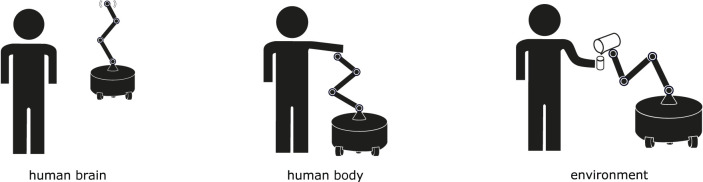
A robot can provide assistance by acting in several different action spaces. It can assist by giving information to the user, adjusting the user’s body, or changing the environment to help complete the task.

To illustrate this point more broadly, we provide a review of recent assistive robotics literature, grouped by whether the robot is acting on the user’s mind, user’s body, or environment (Figure 2).

### 5.1 Environment

One straightforward assistive robot is one that simply completes a task for the user. For example, research has focused on autonomous butler robots (Srinivasa et al., 2010, 2012) that perform tasks such as cooking and cleaning. Such a robot assists a user by navigating around the apartment picking up misplaced items such as dirty laundry and dishes and placing them in appropriate locations such as a laundry hamper or dishwasher. The robot provides assistance by directly changing the environment. To meet the minimal requirement of providing assistance (i.e., delivering some benefit to the target of assistance), the robot must shift the environment from an undesirable state configuration to a more desirable one.

Much research surveyed here assists users in exactly this way: by providing autonomous assistance through environmental state manipulations. Researchers have explored how a user can command a robot to organize a messy room (Mertens et al., 2011; Cremer et al., 2016; Koskinopoulou et al., 2016; Pripfl et al., 2016; Jensen et al., 2017), fetch misplaced or distant items (Iossifidis and Schoner, 2004; Unhelkar et al., 2014; Huang and Mutlu, 2016; Wieser et al., 2016), or even perform more specialized tasks autonomously (under the direction of the user) such as assisted eating (Canal et al., 2016) and other tasks of daily living (Nguyen and Kemp, 2008), search and rescue (Doroodgar et al., 2010), welding (Andersen et al., 2016a), or other industrial tasks (Mueller et al., 2017). Assistive tasks performed autonomously at the request of a user through environmental manipulation can provide several benefits. This method of task execution requires little user input, which makes it efficient for users who prefer not to spend time on chores and beneficial for users who may not be able to accomplish the task at all.

Environmental assistance is not solely the domain of autonomous robots, however. Collaborative robots, specifically in tasks where the user and the robot take independent actions that jointly manipulate the environment towards a mutual goal state, also perform environmental assistance. Examples of such systems include collaborative cleaning (Devin and Alami, 2016) and assembly (Savur et al., 2019; Zhao et al., 2020). A robot working collaboratively with a user can improve its efficiency by modeling the user’s behavior, for example by determining specific poses to hold an object in to facilitate fluid collaboration during assembly (Akkaladevi et al., 2016) or by anticipating and delivering the next required item in assembly (Hawkins et al., 2013, 2014; Maeda et al., 2014) or cooking (Koppula et al., 2016; Milliez et al., 2016), or by providing help under different initiative paradigms during assembly (Baraglia et al., 2016). Collaborative environmental assistance can also be used to perform joint actions with a user, such as in handovers (Cakmak et al., 2011; Kwon and Suh, 2012; Grigore et al., 2013; Broehl et al., 2016; Canal et al., 2018; Cserteg et al., 2018; Goldau et al., 2019; Lambrecht and Nimpsch, 2019; Nemlekar et al., 2019; Newman et al., 2020; Racca et al., 2020), where the goal is to transfer an object from the robot’s end effector to the user’s hand; or co-manipulation (Koustoumpardis et al., 2016; Nikolaidis et al., 2016; Schmidtler and Bengler, 2016; Schmidtler et al., 2016; El Makrini et al., 2017; Goeruer et al., 2018; Rahman, 2019b; DelPreto and Rus, 2019; Rahman, 2020; Wang et al., 2020), where the aim is for the user and the robot to jointly move an object to a specified location or provide redundancy in holding an object in a joint assembly task (Parlitz et al., 2008) or safety critical situation such as surgery (Su et al., 2018).

So far, all examples of environmental assistance have been provided by standalone robots, commonly taking on a humanoid or robotic arm morphology. These robots affect the environment by changing their own configurations first (e.g., using a robot arm to pick up an object). As such, they are considered decoupled from the environment. Robots can also be designed to be coupled with the environment; in these examples, it is hard to distinguish between the robot’s state and the environment state. These robots often take on more conspicuous yet specialized morphologies, such as a mechanical ottoman (Sirkin et al., 2015; Zhang et al., 2018). For example, a robotic suitcase can assist an airline passenger by following them through an airport (Ferreira et al., 2016) and manipulating the user’s sense of trust by moving across various proxemic boundaries. A set of robotic drawers containing tools can assist a user in completing an assembly by proactively opening the drawer containing the next required tool (Mok, 2016), and it can also manipulate a user’s enjoyment in completing the task by employing emotional drawer opening strategies. Environmentally coupled robots can be designed to be “invisible,” (Sirkin et al., 2015) or to be modifications to an existing environment or object. Moving away from more traditional robot appearances may mitigate any negative effects from interacting with a robot.

Other approaches include shared control which separates the responsibilities of the user and the robot during the task. For example a teleoperated surgery robot can hold a patient’s skin taut so that the surgeon can focus on performing incisions (Shamaei et al., 2015). A telepresence robot (Kratz and Ferriera, 2016) can automatically avoid obstacles during navigation (Acharya et al., 2018; Stoll et al., 2018) or automatically rotate its camera to keep a desired object within view (Miura et al., 2016). Finally, a remote, teleoperated space robot can perform as much of a task as is possible before it pings the space station for human intervention (Farrell et al., 2017). By having the robot configure itself according to some of the task requirements, the robot allows the user to focus on other parts of the task.

### 5.2 Human Body

While assistance applied directly to the environment can solve a wide variety of tasks, some tasks require alternate strategies. One such scenario is when some change to the user’s physical state is required to perform the task. For example, consider a robot designed to assist a user who has difficulty bathing themselves. While it is technically possible for that robot to transform the environment by bringing a bathtub to the user, this is obviously impractical. The robot can instead transform the user’s state by bringing them closer to the bathtub (Dometios et al., 2017; Papageorgiou et al., 2019). This strategy of moving a user to assist them is similar to autonomous environmental manipulation, but now the user is being manipulated instead of the environment. This strategy results in limited agency to the user, and is typically only employed when the user has minimal ability to complete the task themselves.

In cases where users can perform some aspects of the task, a robot can also assist by supplementing a user’s existing abilities. For example, if a user can walk but has difficulty balancing or navigating, a smart walker can be utilized to help the user navigate between locations (Papageorgiou et al., 2019; Sierra et al., 2019). Similarly, if a user has some control over their limbs, an exoskeleton robot can be used to provide extra support for day-to-day usage (Baklouti et al., 2008; Lim et al., 2015; Choi et al., 2018; Nabipour and Moosavian, 2018) or in therapeutic scenarios in order to help a user strengthen weakened muscles (Carmichael and Liu, 2013; Zignoli et al., 2019).

In addition to aiding in task execution, physical user state manipulation can also be used to assist in planning, such as when a user’s sensing capabilities are diminished. For example, a visually impaired user may wish to solve a Tangram puzzle but must pick up and feel each piece individually. To provide assistance to the user, a robot could sense the puzzle pieces and determine which pieces are viable for the next step of assembly. The robot can then physically guide the user’s hand to this piece allowing the user to solve the puzzle (Bonani et al., 2018). This is an example of human body state manipulation. Instead of manipulating the environment to solve the task, the robot instead changes the user’s physical state configuration in order to better position them to solve the task.

Robot assistance that acts on a user’s body can also be done by using the resistance of the robot’s own joints. A user kinesthetically manipulating a robot arm, for example, may not know the exact path the arm should travel in order to complete a co-manipulation task. The robot can change its admittance or transparency such that it becomes easier (Jarrasse et al., 2008; Li et al., 2015; Lee and Hogan, 2016; Mariotti et al., 2019; Muthusamy et al., 2019; Luo et al., 2020) or more difficult (Bo et al., 2016; Kyrkjebo et al., 2018; Cacace et al., 2019a,b; Wu et al., 2020) to move as the robot’s end effector deviates from a known, low-cost path. This idea can also be applied to full-scale robots, allowing a user to navigate a robot from one point to another by guiding it as if it were another human (Chen and Kemp, 2010) or to use the stiffness of the robot’s arm as a support while standing up (Itadera et al., 2019). Admittance control as a body state manipulation allows the user to have a high degree of control when operating the robot, but allows the robot to provide information about which parts of the environment are better to traverse by altering the stiffness of its joints. This strategy can also be used in therapeutic settings, where a patient recovering from a stroke can be given an automatic, smooth schedule of rehabilitation exercises as the robot changes its admittance depending on the force feedback it receives from the user (Ivanova et al., 2017).

### 5.3 Human Brain

The final location of assistance we identify is the user’s mental state. These robots assist by transforming the user’s understanding of the world in a helpful way. One common method is for the robot to communicate unknown environmental information to the user. For example, a robot can play particular sounds as it completes its tasks so that a user can track it more easily (Cha et al., 2018). A robot can also describe the local environment for a visually impaired user in a navigation task, enabling them to create a semantic map of the environment (Chen et al., 2016). Similarly, a robot can provide a visual signal to designate objects it intends to interact with so the user can avoid them (Machino et al., 2006; Andersen et al., 2016b; Shu et al., 2018), areas where the robot expects to move so the user can stay away (Hietanen et al., 2019), or areas or paths that the robot thinks the user should take to complete a task in an optimal fashion (Newman et al., 2020). In an emergency scenario, a robot can visually indicate the direction of a safe exit (Robinette et al., 2016). Finally, a robot can provide haptic feedback to indicate when to turn in a navigation task (Moon et al., 2018; Li and Hollis, 2019). Robots that provide alerts like these assist by communicating information about the task or the environment directly to the user so that the user can effectively perform the task.

Robots can also assist in the mental state domain by adopting social roles. Generally, these robots are designed to perform socially beneficial functions similar to those that a human would provide, such as a robot that takes the role of a customer service agent (Vishwanath et al., 2019) or a bingo game leader (Louie et al., 2014). In educational settings such as one-on-one tutoring (Kennedy et al., 2016; Fuglerud and Solheim, 2018; Kanero et al., 2018; van Minkelen et al., 2020) and classroom teaching (Kennedy et al., 2016; Ramachandran et al., 2016; Westlund et al., 2016; Polishuk and Verner, 2018; Ono et al., 2019; Rosenberg-Kima et al., 2019), a robot can deliver lectures in a similar manner to a human teacher. In therapeutic and medical settings, a robot can administer routine medical surveys (Varrasi et al., 2019) independent of the doctor’s social biases (Briggs et al., 2015), provide therapy sessions for routine cognitive behavioral therapy (Dino et al., 2019) or physical therapy (Meyer and Fricke, 2017), and perform other general therapeutic tasks (Agrigoroaie et al., 2016; Fan et al., 2016; Salichs et al., 2018; Alimardani et al., 2020). Finally, a robot’s assistance can vary based on its social role, such as a concierge robot performing different social behaviors when responding to children or adults (Mussakhojayeva et al., 2017), an advice-giving robot providing explanations when a user’s behaviors become non-optimal (Gao et al., 2020) or a robot that gives cooking advice varying its strategies so that the advice is more readily received (Torrey et al., 2013).

Instead of performing a procedure itself, a robot can assist a professional when affecting a user’s mental state. When a therapist is unable to be physically present with a child, for example, a parrot robot can be employed in the home to entice a child with autism to practice skills learned during a therapy session (Shayan et al., 2016; Bharatharaj et al., 2017). During therapy with agitated patients, introducing a pet-like PARO robot can induce mental states more conducive to effective therapy (Shibata et al., 2001; Sabanovic et al., 2013; Chang and Sabanovic, 2015; Shamsuddin et al., 2017). A child-like robot can allow a young patient to practice social skills with a partner more akin to a peer than the therapist is (Goodrich et al., 2011; Kim et al., 2014; Taheri et al., 2014; Ackovska et al., 2017; Nie et al., 2018). Similarly, a child-like robot can assist a teacher by reinforcing a student’s desire to self-engage in educational material, something students may be more likely to learn with a peer than a teacher (Wood et al., 2017; Davison et al., 2020), or increase a user’s ability to recall a story by acting out portions of it (Leite et al., 2015).

Since robot actions are sometimes interpreted socially and as being intentional, robots can select their actions to influence the user’s mental state. For example, predictable and legible motion strategies that indirectly communicate a robot’s goals are readily interpreted by people (Dragan et al., 2013). These same strategies can be used in collaborative tasks to indirectly show the robot’s goal to the user (Bodden et al., 2016; Faria et al., 2017; Zhu et al., 2017; Tabrez et al., 2019). Robots can also mimic human nonverbal behaviors like deictic eye gaze and pointing gestures to indicate task-relevant objects during collaborative tasks (Breazeal et al., 2004; Fischer et al., 2015) or to assist in completing mentally taxing tasks (Admoni et al., 2016; Hemminghaus and Kopp, 2017).

Similarly, robots can use their behavior to suggest their internal emotional state. This strategy can increase rapport, fluidity and reception of a robot’s assistance through emotive motions (Mok, 2016; Terzioglu et al., 2020) or giving the user feedback regarding a task’s success through facial expressions (Reyes et al., 2016; Rahman, 2019a; Christodoulou et al., 2020). Using socially meaningful actions enables assistive robots to communicate with the user efficiently and fluidly.

Robots can also use social behaviors to induce specific, beneficial emotional responses from a user. By mimicking human nonverbal behaviors, robots can use their eye gaze to induce social pressure on a user to work more efficiently (Riether et al., 2012) or to soften its own dominance to allow for better teamwork (Peters et al., 2019). Assistive robotic gestures can also increase feelings of openness in people who are discussing negative experiences (Hoffman et al., 2014) and motivation in users during medical testing (Uluer et al., 2020), in users during physical exercise (Malik et al., 2014; Schneider and Kummert, 2016; Malik et al., 2017), and in stroke patients performing rehabilitative exercises (Tapus et al., 2007). Since people generally view robotic gestures as intentional, robots can use these gestures to induce mental states that assist the user in performing a task.

In addition to nonverbal communication strategies, robots that are capable of speech can converse with users to induce beneficial mental states (Knepper et al., 2017). Robots can use speech to change the content of the conversation (Gamborino and Fu, 2018) or to answer a question about the surrounding environment (Bui and Chong, 2018). Robots can use dialogue to gather information during collaborative teleoperation (Fong et al., 2003), to engender trust in an escape room (Gao et al., 2019), or to facilitate collaboration between two targets of assistance (Strohkorb et al., 2016). Robots can also talk about themselves to influence a user’s view of themselves. For example, tutoring robots for children can make vulnerable statements about themselves to increase trust with the student and student engagement (Martelaro et al., 2016). Similarly, a robot in a group setting can facilitate group trust by leading with vulnerable statements about itself, so that its teammates feel more comfortable sharing their own vulnerabilities. This effect can cascade as more group members explain their own failures, console each other, and laugh together (Sebo et al., 2018). Failing to deliver assistance in contexts where the robot is expected to provide assistance can have deleterious effects on a user’s mental state, causing users to mistrust the robot and harm their relationship and rapport (Kontogiorgos et al., 2020; Rossi et al., 2020).

Beyond focusing on specific content of speech, conversational robots can further affect the user’s mental state in the way they speak. Robots can perform back-channelling to give the appearance of active listening (Birnbaum et al., 2016; Sebo et al., 2020), or give informative feedback to improve task performance (Guneysu and Arnrich, 2017; Law et al., 2017; Sharifara et al., 2018), a user’s self-efficacy (Zafari et al., 2019), or their motivation (Mucchiani et al., 2017; Shao et al., 2019). Robots can choose to only interrupt a distracted user at appropriate times (Sirithunge et al., 2018; Unhelkar et al., 2020). A robot can also change its tone to project an emotion such as happiness to improve the user’s mood and task performance (Mataric et al., 2009; Lubold et al., 2016; Winkle and Bremner, 2017; Rhim et al., 2019). Finally, a robot can combine these qualities with the content of the conversation to change the user’s perception of the robot’s social role (Bartl et al., 2016; Bernardo et al., 2016; Monaikul et al., 2020). Specifically, a robot can act as a student during a tutoring session to induce different learning techniques in a human student (Sandygulova et al., 2020).

Shared control, especially when an input controller (e.g., a joystick) limits the number of input degrees of freedom (Aronson et al., 2018), can also be made easier for user’s by providing assistance that alters the user’s mental state. A robot arm can assist its user by maintaining more easily controllable state configurations (Javdani et al., 2015; Till et al., 2015; Vu et al., 2017; Aronson et al., 2018; Newman et al., 2018) or by optimizing which degrees of freedom the user can control at any given time (Herlant et al., 2016). This idea can be extended to supernumerary arms that provide users with an additional appendage but are difficult to control (Nakabayashi et al., 2018; Vatsal and Hoffman, 2018), teleoperating robotic arms through electromyography (Noda et al., 2013; Pham et al., 2017) or similar sensing devices (Muratore et al., 2019), or humanoid robots (Lin et al., 2019; Zhou et al., 2019). Additionally, a robot might be able to enter environments that are unavailable to a user, allowing the user to teleoperate the robot in these environments, and effectively extend their reachable environment (Horiguchi et al., 2000). These strategies all effectively alter the user’s mental state by decreasing the burden of user communication.

Finally, another strategy for robots to assist a user is by transforming the robot’s own physical configuration into one that is more amenable to task completion. This approach is useful in collaborative scenarios where the robot and user may collide. To avoid this problem, robots can decrease their operating velocity when working in close proximity to users (Araiza-Illan and Clemente, 2018; Rosenstrauch et al., 2018; Svarny et al., 2019) or take paths or actions specifically designed to reduce the likelihood of a collision (De Luca and Flacco, 2012; Hayne et al., 2016; Liu et al., 2018; Nguyen et al., 2018). Similar to shared control, these strategies to assist the user decrease the user’s cognitive burden of planning in the task. By taking responsibility for collisions, a robot can effectively alter its own actions so that the user can be less concerned with monitoring and modelling a robot’s behavior and can concentrate on completing their portion of the task.

### 5.4 Implications

Choosing which action space the robot should act in is a crucial decision for robot designers. To aid users in room cleaning, for example, researchers have developed robots that alter the environment by directly picking up misplaced objects, while others have developed augmented reality solutions that provide assistance in the user’s mental space by showing them routes that, if followed, would lead to the shortest time spent cleaning. Realizing that a given task can be solved by acting in any part of the state allows researchers to develop novel solutions to problems that have historically been restricted to robots that act in a single state.

This realization, however, means that determining the robot’s action space is not simply determined by the task that the robot is being built to solve. Instead, a roboticist must carefully consider the capabilities of the users for whom they are designing the robot. The choice of how the robot acts must be tuned to the needs of the user, and it has broader implications on the user’s sense of agency and trust in the system. This separation of robot action spaces enables designers to compare robots from different domains that have similar action spaces and develop better assistive solutions.

## 6 Time

**FIGURE 3:**
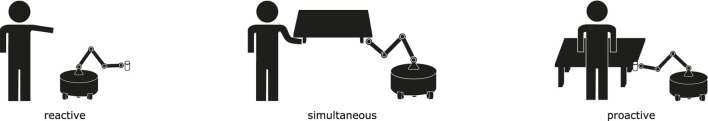
A key axis in assistive robotic systems concerns what type of cue leads to the robot taking actions. Robots can be reactive and respond to explicit input only, be proactive and interpret the general task state to choose to act on their own, or collaborate closely with the user by acting simultaneously with them.

The third key design axis we present concerns how assistive robots coordinate the timing of actions with the targets of their assistance. Consider an assisted eating scenario. A robot might only offer food when given an explicit trigger by the user, or it can monitor the user’s behavior to decide when to initiate the action itself. We categorize the timing of assistive actions as reactive, proactive, or simultaneous. Reactive robots act only when given explicit commands. Proactive robots use predictive models or other approaches to understand the world to initiate their actions without an explicit command. Robots acting simultaneously occur in collaborative settings, during which the robot continuously monitors the user for both explicit and implicit information to direct its actions. Choosing how to time the robot’s behavior can change the difficulty of the task and how users react to the robot’s assistance (Figure 3).

### 6.1 Reactive

Reactive assistance occurs when the assistive action is triggered by an explicit command. Consider a teleoperated robotic arm developed for assistive eating (Javdani et al., 2015; Aronson et al., 2018; Newman et al., 2018). In these studies, a user uses a two-degree of freedom joystick to control a seven-degree of freedom robot arm and pick up a morsel of food from a plate. Direct control of this robot entails only moving the robot’s end-effector while the user is engaging the joystick. The user might also give commands at a higher level of abstraction, perhaps by pressing one button to request food and another for water.

Reactive robots can also respond to more task-specific, contextual triggers. In Canal et al. (2018), an assistive robot helps a user to put on their shoes. This interaction is modeled as a complicated handover problem, where the user must have their foot properly positioned and apply enough resistance that the shoe remains on the foot. In this work, the robot responds to a gesture performed by the user through their foot. When they move their foot in the specified way, the robot knows that it is an acceptable time to place the shoe on their foot.

In general, reactive systems give the user more control over the robot and therefore agency in the overall interaction. Additionally, the robot does not generally need sophisticated models of the task, since it can rely on explicit input from the user. This simplicity means that the robot tends to be less sensitive to the particular task or domain, as it relies on the user to adapt the task to the robot’s capabilities. However, this additional control requires the robot’s user to spend more time and effort on controlling the robot, which can distract from other tasks. Controlling a robot at this level may also require significant training, as the robot’s capabilities may not clearly match the requirements of the task. The control burden grows as the user must explicitly command the robot to begin an interaction (Baraglia et al., 2016), and requiring additional control complexity, such as adding modal control to teleoperation, can be cognitively taxing and slow down progress in the task (Herlant et al., 2016). Furthermore, requiring the user to explicitly cue the robot to act reduces collaborative fluency, which is undesirable as collaborative fluency is a positive attribute that has shown to increase the user’s perceived quality of the interaction (Hoffman et al., 2014) and decrease the time spent during interactions (Huang and Mutlu, 2016).

### 6.2 Proactive

Proactive assistance occurs when the robot predicts that an action would fulfill the user’s goals and takes that action without explicit prompting. For example, in assisted eating, the robot may anticipate a user’s thirst after eating and choose to reach for the glass of water before receiving explicit input. The robot relies on a model of the task and user behavior to estimate what the user would want next. Proactive assistance generally improves the smoothness of interactions, as the assistance target does not need to spend time training or cognitive load to provide explicit instructions to the robot. However, this type of assistance is dependent on the model used to cue its actions, so the added complexity may make the system less reliable.

Consider again the task of operating a high degree of freedom robot using a low degree of freedom input device. Instead of using explicit signals from the user, Herlant et al. (2016) designed a robot that can proactively switch modes. In a simulated navigation task, a user drives a robot whose movement is restricted to exclusively moving either vertically or horizontally through a two-dimensional maze. The robot uses a model of the environment to determine whether horizontal or vertical motion is optimal given the robot’s current position. The robot can then switch the mode proactively, allowing the user to simply direct the robot to move, speeding up the overall interaction time and removing the cognitive burden seen in reactive mode-switching.

Another way a robot can assist proactively is by building a model of the user to infer the task goal before it has been expressed. For example, a robot can predict the next fruit that a customer wants to add to their smoothie (Huang and Mutlu, 2016). Before the user explicitly requests this ingredient, the robot can prepare to grab that ingredient, increasing the fluidity of the interaction.

One challenge of proactive assistance is that users can be uncomfortable or even endangered if the robot makes unexpected motion. To mitigate this concern, the robot can communicate its intentions to the user explicitly. This could be done by having the robot show the user its plan directly on the physical environment, for example highlighting the part of a car door it plans to work on (Andersen et al., 2016b), or by showing its intended travel path in a virtual reality headset (Shu et al., 2018).

Proactive assistance enables more robust and general applications than reactive assistance does. However, the added sophistication in assistance requires additional complexity in the robot’s models and behavior, which is compounded by the need to act in varied environments to unexpected stimuli. In addition, a purely proactive system can be uncomfortable or dangerous if the user is not prepared for the robot’s actions. To mitigate some of these concerns, assistance systems can design some parts of the interaction as reactive and others as proactive. For example, the serving robot in Huang and Mutlu (2016) proactively moves closer to its estimate of the user’s most likely request, but it does not initiate the actual grasping process until it receives an explicit command.

### 6.3 Simultaneous

Simultaneous assistance exists between the previous two categories and includes shared control and collaborative robots. These systems generally function similarly to proactive assistance, but act at the same time as the user. These systems include shared autonomy systems (Javdani et al., 2015; Javdani et al., 2018; Losey et al., 2018), which fuse the user’s direct command with an autonomously generated command and arbitrate between the two according to some schema. It also includes tasks like carrying a table together (Nikolaidis et al., 2016; DelPreto and Rus, 2019), in which both the user and the robot must act independently for progress to be made.

Simultaneous assistance occurs often in collaborative assembly tasks. The goal and structure of a joint assembly task is often pre-specified, making it easy to determine a user’s goal. A robot in such a task can directly assist by, for example, lifting and holding heavy objects steady so that they can be worked on (Fischer et al., 2015; El Makrini et al., 2017). A robot can also assist by orienting a part to optimize construction, for example by following the images found in an assembly manual (Akkaladevi et al., 2016; Wang et al., 2020).

Simultaneous assistance often benefits from sophisticated communication strategies. For example, DelPreto and Rus (2019) designed a robot to sense electromyographic signals from a user to jointly manipulate a heavy object. A robot could also communicate back with the user, for example by changing its stiffness during a co-manipulation task in order to alert the user they should not move an object into a specific location (Bo et al., 2016). Similarly, a robot could provide the user with cues as to the next step during a complicated assembly task such as by pointing at the next item of interest (Admoni et al., 2016), providing a negative emotive feedback when a user completes an incorrect assembly step (Reyes et al., 2016; Rahman, 2019a) or display other emotive capabilities to signal task progress (Mok, 2016; Terzioglu et al., 2020).

Simultaneous assistive systems generally require tight collaboration between the user and the robot. The closeness of the collaboration requires the system to have a more complicated strategy for understanding user commands, since it is unlikely that the user will give precise commands while also accomplishing their task. However, these models can be more flexible than pure proactive systems: the robot can gain immediate feedback from the user about whether or not its action is correct, so it can recover from some model failures more quickly.

### 6.4 Implications

Determining when a robot should act has implications on the quality of a robot interaction. Reactive systems use more explicit control which enables more user agency, but it also increases the burden to complete a task. Proactive systems require more sophisticated models and sensing onboard the robot, but they can improve collaborative fluency while decreasing user burden. Systems that act in anticipation of explicit user commands may even be able to influence future user behavior in unforeseen ways, leading to questions about who is in control of setting the task goal (Newman et al., 2020). Proactive robots also generally lead to more robot agency, which introduces complex challenges such as safety and trust.

Preferences among when a robot chooses to take action may differ among users even within the same task domain. While one user may prefer a robot that requires less training and complication to operate, another might prefer to have more direct control over the robot to determine its behavior more precisely. If the user is paired with the system they least prefer, the interaction may cease to be assistive. In addition, an assistive system need not be completely proactive, reactive or simultaneous: the system can choose different timing and cueing strategies based on the particular part of the task under consideration. Choosing exactly when a robot executes its actions requires careful thought about the nature of the task, the capability of the robot, and the desires of the user.

## 7 Conclusion

In this paper, we describe an overall perspective on robotic systems that emphasizes their assistive intentions. With this perspective, we present three key design axes that compare assistive robotics research across domains: the relationships they develop with people, their action space, and their action timing. We explore these axes through a review of recent assistive robotics research, showing how assistive robots from across domains face similar challenges and make comparable decisions along these axes.

Much of the research discussed in this paper is specific to its task domain due to how the field has been organized and the difficulty of building abstractions. In this work, we propose some abstractions, and we hope that they will enable designers of assistive robots to find systems in other domains that share their problems and to draw deeper connections with them.

For each axis, we discuss design tradeoffs resulting from particular approaches. From among these axes, several themes emerge. Choices in the robot’s action space and timing can both affect a user’s sense of agency. Similarly, both the robot’s action space and relationship with the user impact the structure of the communication between the robot and the user, which alters the quality of the assistance. It is our hope that researchers will explore more themes that span these design axes and provide more structure to the development of assistive robots.

Finally, this work is intended to start a conversation about how to understand the specific challenges of assistive robotics within the general area of human-robot interaction. With this framework, we hope to encourage researchers to further explore the nature of assistance as a general concept and describe its inherent challenges. We do not claim that these axes are complete; rather, we present them as the beginning of a larger effort to develop general principles of assistive robotics.
